# Complete genome sequences of bacteriophages Crescenzo, Roberts, and Gerri43 isolated from *Arthrobacter globiformis*

**DOI:** 10.1128/mra.00956-26

**Published:** 2026-09-29

**Authors:** Gabrielle Achinko, Maximillian Castro, Evan Chernyenko, Kelley Crescenzo, Natalie Horchuck, Jessica Lee, Mattie McMahon, Heidi Meehan, Sandra Orjih, Kanishka Poudel, Kaitlyn Roberts, Alex Bogush, Matthew Farber, Marina Bogush

**Affiliations:** 1 Rowan University College of Science and Mathematics 367268, Glassboro, New Jersey, USA; 2 University of Pittsburgh Swanson School of Engineering 110071, Pittsburgh, Pennsylvania, USA; Queens College Department of Biology, Queens, New York, USA

**Keywords:** *Arthrobacter globiformis*, bacteriophages, sequence analysis, genomics

## Abstract

We report the complete genome sequences of the *Arthrobacter globiformis* B-2979 phages Crescenzo, Roberts, and Gerri43. Crescenzo contains unique genes without recognizable homologs, while Roberts and Gerri43 lack a ribonucleotide reductase gene. Roberts genes *52* and *61* lack conserved stop codons, resulting in extended predicted gene products.

## ANNOUNCEMENT

Antimicrobial resistance caused an estimated 1.27 million deaths in 2019, highlighting the need for alternative treatments such as bacteriophage (phage) therapy (1, 2). Phages may also improve food safety by reducing pathogenic bacteria through phage biocontrol (3).

**Fig 1:**
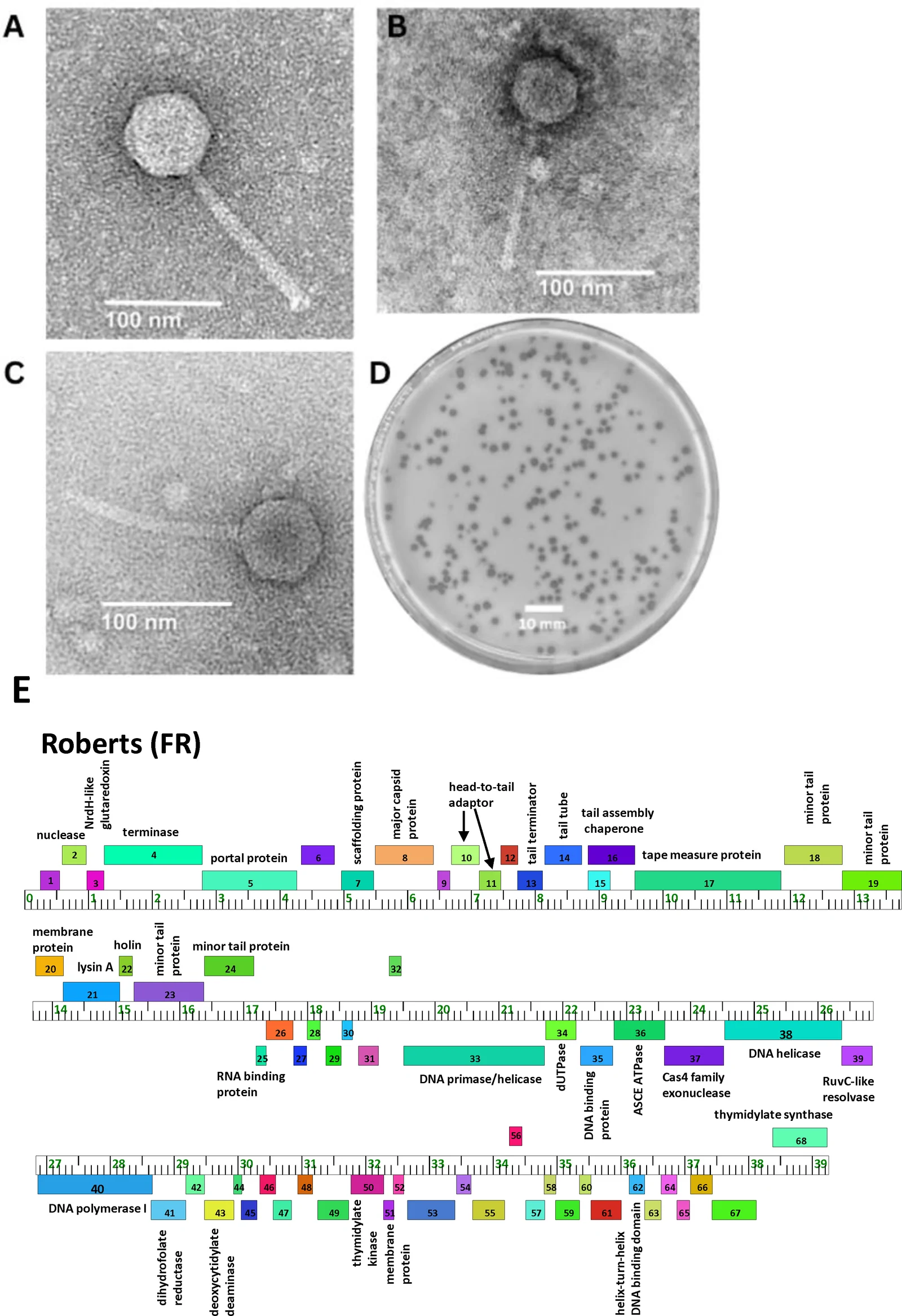
Characterizations of *Arthrobacter globiformis* phages Roberts, Crescenzo, and Gerri43. (**A–C**) Roberts, Crescenzo, and Gerri43 virion particle morphology was determined by transmission electron microscopy (TEM). TEM was performed at the University of Maryland using phage lysates that were negatively stained with 1% uranyl acetate and applied to Formvar film-coated, carbon Type-B, 300-mesh copper grids. Images were obtained using Hitachi HT7800 TEM 100 kV accelerating voltage and 60× magnification. (**D**) Plaque assay of Crescenzo. Plaques measure 1–3 mm in diameter (*n* = 12). Plaques by phages Roberts and Gerri43 form plaques of similar morphology (data not shown). Images of phage plaques were captured using the constructed IllumiPlate device, which provides uniform, diffuse lighting for visualization and analysis of phage plaques. (**E**) Phamerator map of phage Roberts showing predicted size, position, and function of 68 genes. The map was generated using Phamerator with database Actino_Draft_579 (5).

Bacteriophages Crescenzo and Roberts were isolated from soil samples collected on the Rowan University campus in Glassboro, NJ, USA, and Gerri43 from a soil sample collected in Greenwich, NJ, USA. Soil samples were suspended in PYCa medium (30 mL), shaken for 1 h, centrifuged, and filtered (0.22 μm) to isolate bacteriophages. The filtrates were enriched with *Arthrobacter globiformis B-2979* (500 μL) for 48 h at 30°C and then filtered and plated in PYCa top agar with *A. globiformis* (4). Isolated plaques were purified through three rounds of plating to ensure clonal phage populations, and uranyl acetate-stained transmission electron microscopy (TEM) revealed siphoviral morphologies for all three phages (Fig. 1A through D).

DNA was isolated from the phage lysates using the Norgen Phage DNA Isolation Kit, prepared for sequencing using the NEBNext Ultra II Library Prep Kit, and sequenced on an Illumina NextSeq 1000 (XLEAP-P1 kit) (Table 1). Raw reads were trimmed with cutadapt 4.7 (using the option: -nextseq-trim 30) and filtered with skewer 0.2.2 (using the options: -q 20 -Q 30 -n -l 50) prior to assembly with Unicycler and finishing with Consed (6–9). Based on ≥35% gene content similarity to phages in the Actinobacteriophage database (PhagesDB), Crescenzo was assigned to cluster AY, while Roberts and Gerri43 were assigned to cluster FR (10–12).

**TABLE 1:** Characterizing the genomic contents of *Arthrobacter globiformis* bacteriophages Crescenzo, Gerri43, and Roberts^*a*^

Parameter	Crescenzo	Roberts	Gerri43
Number of 100 bp reads	495,525	705,407	687,248
Approximate shotgun coverage (×)	151	198	200
Genome length (bp)	53,361	39,638	39,638
Genome end type	3′ single-stranded overhang	3′ single-stranded overhang	3′ single-stranded overhang
Overhang length	9 bases	10 bases	10 bases
GC content	62.4%	58.9%	58.9%
Cluster	AY	FR	FR
Collection date	9/13/25	9/14/25	9/14/25
Collection coordinates	39.711667 N, 75.122222 W	39.70833 N, 75.1145 W	39.89386 N, 75.34353 W
Tail length	112.0 ± 2.8 nm (*n* = 2)	100.5 ± 0.7 nm (*n* = 2)	124.5 ± 0.7 nm (*n* = 2)
Capsid length	62.5 ± 2.1 nm (*n* = 2)	46.5 ± 0.7 nm (*n* = 2)	61.25 ± 0.35 nm (*n* = 2)
Total number of identified genes	94	68	68
Number of genes with identified functions	52	35	33
Number of genes without identified functions	42	33	35
Number of tRNA genes	1	0	0
GenBank accession	PZ492779	PZ575429	PZ492786
SRA accession	SRX32199481	SRX32199483	SRX32199482

^*a*^ Phage DNA concentrations were quantified using a qubit fluorometer. The extracted DNA concentrations were 20 ng/µL for Roberts, 103 ng/µL for Gerri43, and 22 ng/µL for Crescenzo, with total recovered DNA volumes of 185, 70, and 70 µL, respectively. Approximately 400 ng of DNA from each sample was used for sequencing library preparation.

Glimmer v3.02 and GeneMark v2.5 were used for auto-annotation, followed by manual refinement with Phamerator v580, Starterator v658 (http://phages.wustl.edu/starterator), and PECAAN (5, 13–15). Functional assignments were evaluated using BLASTp v 2.2.26 (using the National Center for Biotechnology Information [NCBI] nonredundant database and the database at phagesdb.org) and HHpred (using PDB_mmCIF70, PfamA, and NCBI Conserved Domain databases [16, 17]). A single tRNA-Phe(GAA) gene (Crescenzo) was identified using Aragorn v1.2.38 and tRNAscan-SE v2.0 (18, 19; Table 1). Default settings were used for all programs.

Comparison with cluster AY phages (20) revealed several Crescenzo genes without recognizable homologs, including genes *33* and *41*, located near predicted tyrosine integrase (*38*) and near the genome end (*94*).

BLASTn (16) analysis showed that the Roberts (Fig. 1E) and Gerri43 share 99.95% nucleotide identity, differing by 19 nucleotides. Among these differences is the absence of a conserved stop codon in Roberts gene *52*, resulting in a predicted gene product approximately 20 amino acids longer than its corresponding protein in other FR phages. A similar absence of a conserved stop codon occurs in Roberts gene *61*, resulting in the extended predicted gene product. Comparison of FR phages (21) showed the greatest genomic divergence at the genome ends, where many genes have not been assigned putative functions, including a ribonucleotide reductase gene that is absent in Roberts and Gerri43.

## Data Availability

The data generated in this study are available in GenBank/SRA under accession numbers PZ492779/SRX32199481 (Crescenzo), PZ575429/SRX32199483 (Roberts), and PZ492786/SRX32199482 (Gerri43).
